# 实时肛拭子Xpert Carba-R检测预警造血干细胞移植患者CRO血流感染的临床可行性研究

**DOI:** 10.3760/cma.j.cn121090-20240607-00213

**Published:** 2024-11

**Authors:** 岱蓉 谢, 晨静 千, 紫璇 李, 威 石, 照东 仲, 凌辉 夏, 秋玲 吴, 梅 洪

**Affiliations:** 1 华中科技大学同济医学院附属协和医院血液科，武汉 430022 Department of Hematology, Union Hospital, Tongji Medical College, Huazhong University of Science and Technology, Wuhan 430022, China; 2 河南科技大学第一附属医院血液科，洛阳市血液病移植免疫重点实验室，洛阳 471000 Department of Hematology, The First Affiliated Hospital of Henan University of Science and Technology, Key Laboratory of Transplantation Immunology for Haematological Diseases, Luoyang 471000, China

**Keywords:** 造血干细胞移植, 耐碳青霉烯类革兰阴性菌, 肠道菌群, 定植, 血流感染, Xpert Carba-R, Hematopoietic stem cell transplantation, Carbapenem-resistant organism, Intestinal microbiota, Colonization, Bloodstream infection, Xpert Carba-R

## Abstract

**目的:**

分析造血干细胞移植（HSCT）患者耐碳青霉烯类革兰阴性菌（CRO）肠道定植菌株与血流感染（BSI）菌株的同源性，验证实时肛拭子Xpert Carba-R检测在临床应用中的价值，探讨其预警BSI的可行性。

**方法:**

收集并分析2021年1月至2021年12月从HSCT患者的肛拭子和血培养样本中获得的耐药菌株，使用菌种鉴定、抗菌药物耐药表型、全基因组测序（WGS）、多位点序列分型和碳青霉烯酶型鉴别等方法，验证CRO肠道定植和BSI菌株的同源性。对2021年8月至2022年8月的HSCT患者同时进行肛拭子培养和实时肛拭子Xpert Cabar-R酶型筛查，以纯化菌落碳青霉烯酶基因多重实时聚合酶链反应（PCR）扩增产物的测序结果为参照标准，验证实时肛拭子Xpert Cabar-R检测的准确性。

**结果:**

本研究共纳入10例HSCT患者的24株CRO菌株，其中肠道定植菌14株，BSI菌10株。同一患者的CRO肠道定植和BSI菌株及其碳青霉烯酶基因高度一致，WGS显示同一患者的CRO肠道定植菌和BSI菌的亲缘关系比不同患者的菌株关系更密切。此外，本研究纳入184例HSCT患者的488份肛拭子标本，肛拭子培养和实时肛拭子Xpert Cabar-R的CRO检出率分别为16.4％和18.4％，实时肛拭子Xpert Carba-R检测的总体敏感性、特异性、阳性预测值和阴性预测值分别为96.6％、72.8％、90.6％和88.9％。

**结论:**

HSCT患者CRO肠道定植菌株与BSI菌株具有高度同源性，实时肛拭子Xpert Carba-R是检测常见碳青霉烯酶基因可靠且方便的方法，可替代肛拭子培养预警CRO-BSI的发生。

## Abstract (en)

**Objective:**

This study aimed to analyze the homology between carbapenem-resistant organisms（CRO）intestinal colonization strains and bloodstream infection（BSI）strains in patients undergoing hematopoietic stem cell transplantation（HSCT）, confirming the clinical use of the real-time rectal swab Xpert Carba-R assay, and investigate its feasibility in early warning of BSI.

**Methods:**

Drug-resistant strains obtained from rectal swabs and blood culture samples of patients undergoing the same HSCT from January 2021 to December 2021 were collected and analyzed. The homology of the CRO intestinal colonization and BSI strains was confirmed using strain identification, antimicrobial resistance phenotyping, whole genome sequencing（WGS）, multilocus sequence typing（MLST）, and carbapenemase type identification. Rectal swab cultures and the real-time rectal swab Xpert Carba-R assay were conducted concurrently on patients with HSCT from August 2021 to August 2022. The accuracy of the real-time rectal swab Xpert Carba-R assay was confirmed with the sequencing results of polymerase chain reaction amplification products of the carbapenemase gene from purified colonies as a reference standard.

**Results:**

This study included 24 CRO strains from 10 patients undergoing HSCT, including 14 intestinal colonizers and 10 CRO-BSI strains. The results revealed that the CRO intestinal colonization strains and CRO-BSI strains from the same patient and their carbapenemase genes were almost identical. Additionally, WGS revealed that CRO intestinal colonization and CRO-BSI strains from the same patient were more closely related than strains from different patients. Additionally, this study included 488 rectal swab specimens from 184 patients undergoing HSCT, with CRO detection rates of 16.4％ for rectal swab culture and 18.4％ for the real-time rectal swab Xpert Carba-R assay. The overall sensitivity, specificity, and positive and negative predictive values of the real-time rectal swab Xpert Carba-R assay were 96.6％, 72.8％, 90.6％, and 88.9％, respectively.

**Conclusion:**

A high degree of homology was found between the CRO intestinal colonization strains and the CRO-BSI strains in patients undergoing HSCT. The real-time rectal swab Xpert Carba-R assay is a reliable and convenient method for detecting common carbapenemase genes, serving as an alternative to rectal swab culture for early warning of CRO-BSI.

耐碳青霉烯类革兰阴性菌（CRO）引起的感染日趋严峻，病死率高，医疗负担严重[1]。恶性血液病患者是发生CRO感染的高危人群，较其他患者有更高的肠道定植率、感染率和病死率[2]–[3]。相较于其他感染部位，恶性血液病患者更容易发生CRO血流感染（BSI），30 d相关病死率高达51％，患者从出现症状到死亡的中位时间仅为96 h[4]–[5]。目前血培养是诊断CRO-BSI的金标准，但由于其阳性率低且检测时间长，获得结果的平均时间为24～48 h，从而导致及时有效的抗感染治疗延迟，极大增加了患者的感染死亡率[6]。早期识别CRO相关感染至关重要，本研究旨在寻找预警CRO-BSI的有效措施。

肛拭子培养已常规用于CRO肠道定植的检测[7]，恶性血液病患者存在CRO定植是CRO-BSI发生的主要影响因素，CRO定植的造血干细胞移植（HSCT）患者发生CRO-BSI的概率（50％）显著高于未定植患者（7.5％）[8]。因此，CRO肠道定植可能预警CRO-BSI的发生。实时肛拭子Xpert Carba-R（美国Cepheid公司产品）是一种检测碳青霉烯酶的技术，采用聚合酶链反应（PCR）在不到1 h内检测5种常见的碳青霉烯类水解酶基因KPC、NDM、VIM、IMP和OXA-48，从而快速鉴定出菌株的耐药机制[9]–[10]。本研究通过多种方法分析CRO定植菌株与BSI菌株的同源性，验证实时肛拭子Xpert Carba-R检测在HSCT患者中的临床效能，探讨其预警HSCT患者发生CRO-BSI的临床可行性。

## 病例与方法

1. 病例：2021年1月至2021年12月在华中科技大学同济医学院附属协和医院血液科每周接受肛拭子筛查的HSCT患者，一旦CRO肛拭子培养阳性（定植菌株）后出现感染症状（体温≥37.4 °C、感染指标异常等），立即采集血培养以判定是否发生CRO-BSI。使用包括菌种鉴定、抗菌药物耐药表型、多位点序列分型（MLST）、全基因组测序（WGS）和碳青霉烯酶型鉴别等在内的多种方法测定CRO肠道定植菌株与CRO-BSI菌株的同源性。如果同一患者在住院期间有≥2次血培养CRO阳性，只选择纳入第一次BSI时的相关资料。

2021年8月至2022年8月HSCT患者每周均进行CRO肛拭子筛查。同时收集每例患者2份肛拭子标本，其中1份进行肛拭子（rectal swab, RS）传统培养（RS-培养组），另1份进行实时肛拭子Xpert Carba-R检测（实时RS-Carba-R组）。一旦发现RS-培养组和（或）实时RS-Carba-R组出现CRO阳性，将CRO纯化菌落进行Xpert Carba-R检测（纯化菌落Xpert Carba-R），以进一步确认是否存在碳青霉烯酶基因及其类型，并将CRO纯化菌落储存在−80 °C冰柜中以备后续复测，验证Xpert Carba-R检测方法与标准方案的临床等效性。本研究已通过华中科技大学同济医学院附属协和医院伦理委员会批准，伦理批号：[2020]伦审字（0572）号。

2. 肛拭子标本采集：将肛拭子插入肛门括约肌3～5 cm处到达位于齿状线上的直肠远端，轻轻旋转取出，置于运输培养基中，送至微生物实验室进行菌株培养与鉴定。

3. 菌株鉴定及药敏试验：病原菌的分离鉴定严格按照国家临床实验室规程的相关规定进行。使用Vitek® 2自动化系统及基质辅助激光解吸电离飞行时间质谱等方式进行细菌鉴定和药物敏感性测试。采用圆盘扩散法（K-B法）和微量肉汤稀释法检测抗菌药物的最小抑菌浓度（Minimal inhibitory concentration, MIC）。除替加环素和多黏菌素外，所有抗生素均根据临床和实验室标准协会（Clinical and Laboratory Standards Institute, CLSI）M100S 28th标准进行判读。多黏菌素和替加环素的药敏结果则按照欧洲抗菌药物敏感性试验委员会（European Committee on Antimicrobial Susceptibility Testing, EUCAST）的标准。

4. 肛拭子Xpert Carba-R检测：①预处理：将单头肛拭子样本取出后沿样品试剂瓶口边缘折断，使拭子能够放入瓶中，拧紧瓶盖并高速涡旋混合10 s；②上样：打开样本检测盒的盖子，使用提供的移液管从样本试剂瓶中吸取约1.7 ml制备好的样本，然后将吸取的液体加入Xpert Carba-R样本检测盒中；③上机检测：关闭样本检测盒的盖子，最好在样本加入后30 min内将检测盒放入Xpert Carba-R仪器中进行检测，约1 h后读取结果。

5. Xpert Carba-R试验的临床等效性评估：①结果判断：阳性结果是试剂盒检测到至少一种靶标基因，阴性结果是试剂盒未检测到任何一种靶标基因。②不一致性试验：当实时肛拭子Carba-R试剂盒检测结果呈阴性，而纯化菌落碳青霉烯酶基因测序结果呈阳性时，需补充纯化菌落PCR；当实时肛拭子Carba-R试剂盒检测结果呈阳性，而纯化菌落碳青霉烯酶基因测序结果呈阴性时，需补充CRO重复培养基PCR（表1）。③数据统计分析：以纯化菌落碳青霉烯酶基因PCR扩增产物的测序结果为参照标准，判断实时肛拭子Carba-R检测结果的真阳性、假阳性、真阴性、假阴性，其中假阳性结果通过不一致性试验结果补充矫正。灵敏度＝真阳性/（真阳性+假阴性）×100％；特异度＝真阴性/（真阴性+假阳性）×100％；阳性预测值（PPV）＝真阳性/（真阳性+假阳性）×100％；阴性预测值（NPV）＝真阴性/（假阴性+真阴性）×100％。

**表1:** Xpert Carba-R试验的临床等效性评估表

实时肛拭子Carba-R	纯化菌落Xpert Carba-R	不一致性试验
阴性	阳性	纯化菌落PCR
阳性	阴性	CRO重复培养基PCR

**注** CRO：耐碳青霉烯类革兰阴性菌

6. 碳青霉烯酶基因PCR检测：PCR法检测待测菌株常见碳青霉烯酶基因（blaKPC、blaNDM、blaVIM、blaIMP、blaOXA-48）。

（1）脱氧核糖核酸（DNA）的提取：将保存于−80 °C的菌株取出，划线接种于血液琼脂培养基，37 °C培养24 h后，挑取饱满菌落，置于装有200 µl无菌水的EP管中充分混匀；EP管置于100 °C金属浴10 min后冷却至室温，13 000 r/min（离心半径10 cm）离心5 min后，将上清液转移至新的1.5 ml无菌管中，可于−20 °C短期保存备用。

（2）耐药基因检测：PCR引物序列见表2。

**表2:** 耐药基因PCR引物序列

基因	序列（5′→3′）	扩增片段长度（bp）	退火温度（°C）
blaKPC	F-ATGTCACTGTATCGCCGTCT	893	60
	R-TTTTCAGAGCCTTACTGCCC		
blaNDM	F-GAATTCGCCCCATATTTTTGC	977	59
	R-AACGCCTCTGTCACATCGAAAT		
blaOXA-48	F-GCGTGGTTAAGGATGAACAC	438	58
	R-CATCAAGTTCAACCCAACCG		
blaIMP	F-CTACCGCAGCAGAGTCTTTG	588	55
	R-AACCAGTTTTGCCTTACCAT		
blaVIM	F-TTATGGAGCAGCAACGATGT	621	55
	R-CGAATGCGCAGCACCAGG		

（3）PCR扩增：①扩增条件如下：95 °C预变性3 min；98 °C变性15 s，53 °C退火15 s，72 °C延伸15 s，循环30次；72 °C延伸5 min；4 °C终止反应。②琼脂糖凝胶电泳。③提取模板DNA待测菌株经血液琼脂培养基36 °C培养18 h后，取单个菌落于装有150 µl纯水的无菌离心管中制成菌悬液，于100 °C水浴中煮沸15 min，冷却后，12 000 r/min（离心半径10 cm）离心2 min，取上清液（模板DNA）−20 °C保存待用。

7. 全基因组测序：将上述10例患者的24株CRO菌株送至武汉臻熙医学检验实验室有限公司高通量测序服务组完成二代全基因组测序，核酸提取样本的测序过程主要包括文库构建、扩增测序、质控三个主要步骤。

8. 二代测序及数据分析：每份样本取1 µl，使用Qubit®荧光仪定量检测提取核酸浓度，定量使用的试剂盒为Qubit dsDNA HS Assay Kit；根据核酸浓度，每份样本取500 ng总量，于武汉臻熙实验公司进行文库构建及二代测序。

（1）菌株鉴定：使用StrainSeeker（https://bioinfo.ut.ee/strainseeker）进行菌株鉴定[11]。

（2）MLST测定：使用Linux系统运行MLST软件，对细菌基因组的7个管家基因gapA、infB、mdh、pgi、phoE、rpoB和tonB进行对齐和分类，每个基因组的每种管家基因被命名为1个随机整数，各种管家基因命名整数的唯一组合（等位基因谱）即为一种序列类型（Sequence types，ST），最后根据细菌ST初步分析其同源性。

（3）菌株进化分析：基于单核苷酸多态性（Single nucleotide polymorphism，SNP）方法对收集的肺炎克雷伯菌和大肠埃希菌进行分析。于美国国家生物技术信息中心（NCBI）下载肺炎克雷伯菌和大肠埃希菌的参考基因组，使用bwa（version 0.7.17-r1188）进行参考基因组比对，然后使用freebayes（version v1.0.2）进行SNP检测，最后使用MEGA（version 11.0.13）通过极大似然估计构建进化树。

（4）耐药基因鉴定：运用ResFinder软件（version 4.2.4）[12]及ResFinder数据库对细菌进行耐药基因鉴定和聚类热图。

9．统计学处理：通过比较实时RS-Carba-R组结果与复合诊断（纯化菌落Xpert Carba-R和补充测试）结果验证实时肛拭子Xpert Carba-R检测的灵敏度、特异度、PPV和NPV。连续变量的比较应用Student *t*检验（正态分布变量）或Mann-Whitney *U*检验（非正态分布变量），符合正态分布的定量资料用*x*±*s*表示，符合偏态分布的定量资料用*M*（范围）表示，此外，分类变量用例数（百分比）表示。使用SPSS 25.0软件进行统计学分析。

## 结果

1. CRO肛拭子定植菌株和BSI菌株的同源性：

（1）菌株同源性：在本研究中，共有10例患者的24株CRO菌株最终被纳入研究，其中肠道CRO定植菌14株，CRO-BSI菌10株。使用包括菌种鉴定、抗菌药物耐药表型、MLST、WGS和碳青霉烯酶型鉴别等在内的多种方法对上述24株CRO菌株进行测定。

传统菌株培养和鉴定结果见表3，24株CRO菌株类型分别为肺炎克雷伯菌（15株）、变栖克雷伯菌（2株）、大肠埃希菌（5株）、阴沟肠杆菌（2株）。每例患者CRO-BSI菌株和肠道定植菌株之间的肺炎克雷伯菌（例1、3、5、6、9、10）、大肠埃希菌（例7、8）、阴沟肠杆菌（例2）和变栖克雷伯菌（例4）高度一致。实验室的传统菌株培养和鉴定显示，患者的CRO肠道定植菌株和CRO-BSI菌株具有相同的表型。为了更好地鉴定CRO-BSI菌株来源，进一步通过Strain Seeker（一种从原始测序读段中检测细菌菌株的工具）检验该假设（表3）。分析结果显示，肺炎克雷伯菌CAV1392是最常见的菌株，来自同一患者的CRO肠道定植菌株和CRO-BSI菌株具有一致性（表3）。

**表3:** 10例患者24株CRO肛拭子定植菌株和血流感染菌株的组成及序列型

例号	菌株号	标本来源	菌株组成（Strain Seeker）	序列型
1	1	肛拭子培养	肺炎克雷伯菌菌株DMC1097	1128
	2	血培养	肺炎克雷伯菌菌株DMC1097	1128
2	3	肛拭子培养	阴沟肠杆菌菌株ECNIH5	78
	4	血培养	阴沟肠杆菌菌株ECNIH5	78
3	5	肛拭子培养	肺炎克雷伯菌菌株CAV1392	11
	6	血培养	肺炎克雷伯菌菌株CAV1392	11
4	7	肛拭子培养	变栖克雷伯菌菌株DSM 15968	新序列型
	8	血培养	变栖克雷伯菌菌株DSM 15968	新序列型
5	9	肛拭子培养	肺炎克雷伯菌菌株CAV1392	11
	10	血培养	肺炎克雷伯菌菌株CAV1392	11
6	11	肛拭子培养	肺炎克雷伯菌菌株CAV1392	11
	12	血培养	肺炎克雷伯菌菌株CAV1392	11
7	13	肛拭子培养	大肠埃希菌菌株SE11 DNA	156
	14	血培养	大肠埃希菌菌株SE11 DNA	156
	15	肛拭子培养	大肠埃希菌菌株SE11 DNA	156
8	16	肛拭子培养	大肠埃希菌菌株6409	新序列型
	17	血培养	大肠埃希菌菌株6409	新序列型
9	18	肛拭子培养	肺炎克雷伯菌菌株CAV1392	11
	19	血培养	肺炎克雷伯菌菌株CAV1392	11
	20	肛拭子培养	肺炎克雷伯菌菌株CAV1392	11
10	21	肛拭子培养	肺炎克雷伯菌菌株CAV1392	11
	22	肛拭子培养	肺炎克雷伯菌菌株CAV1392	11
	23	血培养	肺炎克雷伯菌菌株CAV1392	11
	24	肛拭子培养	肺炎克雷伯菌菌株CAV1392	11

**注** CRO：耐碳青霉烯类革兰阴性菌

将上述14株CRO肛拭子定植菌和10株CRO-BSI菌株的全基因组序列上传至PubMLST数据库进行分型比对，其MLST分型结果见表3。分析结果显示，来自例3、5、6、9、10的13株菌的ST均为11。与PubMLST数据库中的数据进行比较显示，来自例4和例8的4个ST均为新ST，此前未被报道。

将样本中CRO肠道定植菌株和CRO-BSI菌株的系统发育相关性进行比较。基于SNP的系统发育分析表明（图1），同一患者的CRO肠道定植菌株和CRO-BSI菌株的亲缘关系较无关患者更近，同一患者的CRO肠道定植菌株与CRO-BSI菌株高度同源。来自同一患者的肺炎克雷伯菌中，CRO肠道定植菌株和CRO-BSI菌株之间平均只有166个单核苷酸变异（Single nucleotide variants, SNV）（每兆碱基30个SNV），而在来自不同患者的菌株之间检测到26 975个SNV。对于大肠埃希菌，CRO肠道定植菌株和CRO-BSI菌株之间SNV的平均值为1 835（每兆碱基375个SNV），不同患者之间为44 028个SNV。

**图1:**
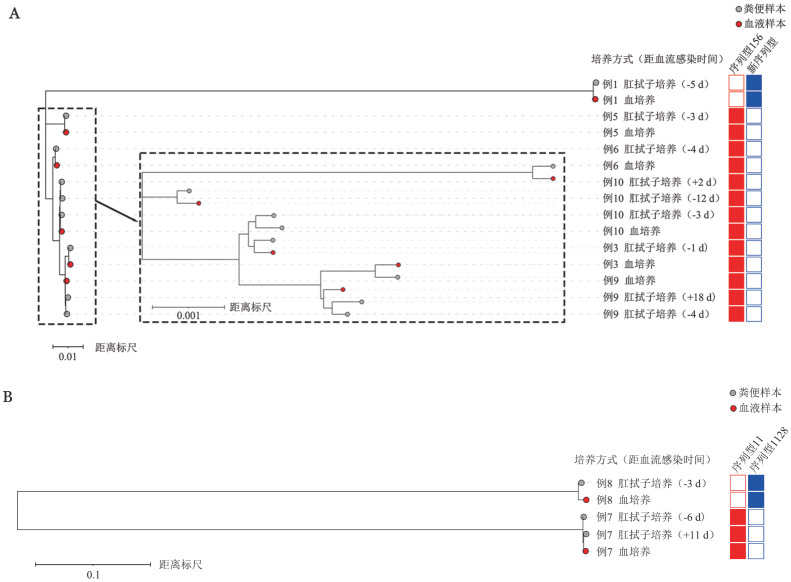
基于肺炎克雷伯菌（A）和大肠埃希菌（B）的单核苷酸多态性系统发育树

（2）碳青霉烯酶基因的一致性：通过WGS鉴定上述24株CRO菌株所携带的碳青霉烯酶基因，比较每例患者CRO-BSI菌株与肠道定植菌株之间碳青霉烯酶基因的一致性。结果显示，碳青霉烯酶基因类型包括KPC（13株）、NDM（7株），而来源于例1和例4的肛拭子CRO定植菌和CRO-BSI菌株最终鉴定为不携带碳青霉烯酶基因。同一患者的BSI和肛拭子CRO菌株中的碳青霉烯酶基因高度一致（例7、8的大肠埃希菌均携带NDM；例3、5、6、9、10的肺炎克雷伯菌均携带KPC）。

为了鉴定上述14株CRO肠道定植菌株和10株CRO-BSI菌株所携带的不同耐药模式，基于WGS进行了耐药基因分层聚类分析（图2），来自同一患者的CRO肠道定植菌株和CRO-BSI菌株的抗生素耐药特征相似。分析表明，肺炎克雷伯菌和大肠埃希菌具有多种抗生素耐药基因，对多种抗生素耐药。肺炎克雷伯菌（13株）携带blaKPC-2基因，所有大肠埃希菌（5株）均携带blaNDM-5基因。

**图2:**
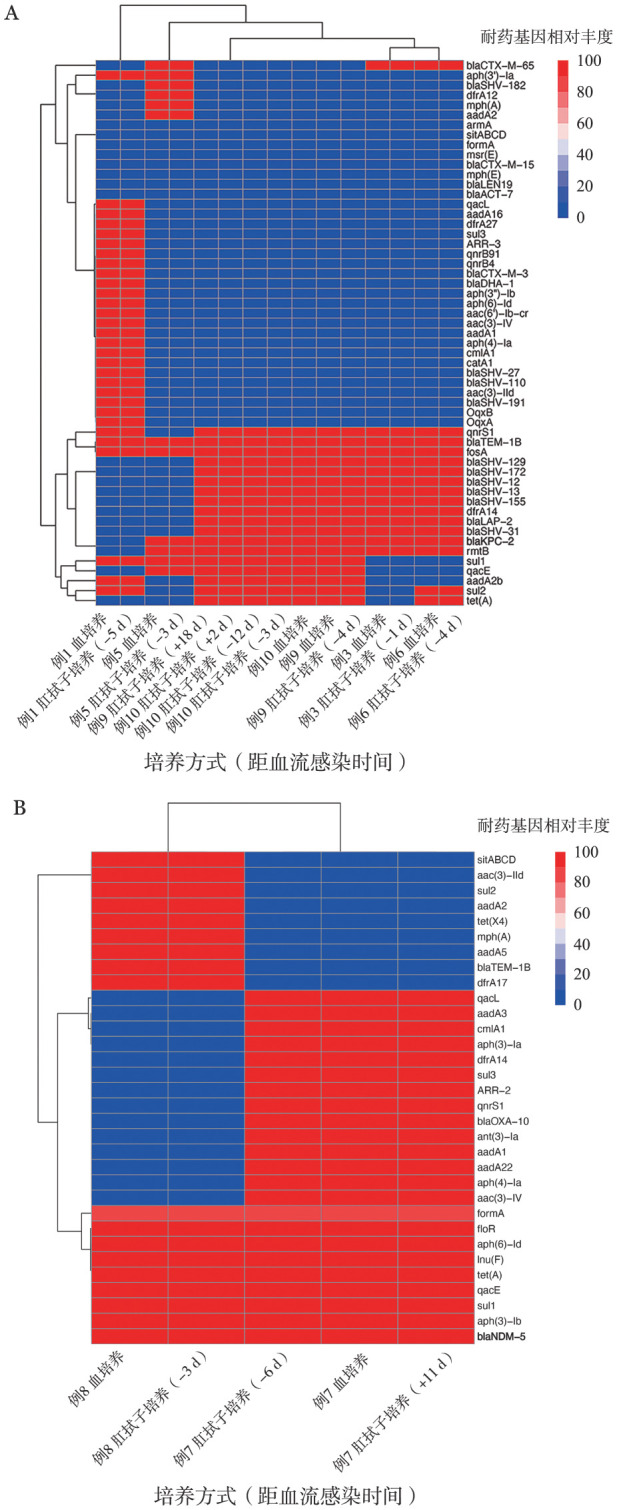
基于全基因组测序的肺炎克雷伯菌（A）和大肠埃希菌（B）的耐药基因分层聚类分析热图 **注** 红色：基因存在；蓝色：基因缺失

2. 实时肛拭子Xpert Carba-R检测的准确性：

（1）微生物学和碳青霉烯酶基因特征：2021年8月至2022年8月，184例HSCT患者进行了针对CRO的RS培养和实时RS-Carba-R筛查。其中男105例（57.1％），≤45岁123例（66.8％），急性髓系白血病60例（32.6％），为主要原发疾病。162例（88.0％）患者接受了allo-HSCT，122例（66.3％）HSCT患者HLA不相合。

研究期间，从184例患者中共采集488份临床肛拭子标本，RS-培养组和实时RS-Carba-R组的CRO检出率分别为16.4％和18.4％，总CRO定植率为22.3％（41/184）。RS-培养组共检测到36株CRO分离株，其中检出最多的CRO菌种为大肠埃希菌（11/36，30.6％），其次是阴沟肠杆菌（7/36，19.4％）、肺炎克雷伯菌（4/36，11.1％）和奇异变形菌（3/36，8.3％）。此外，还检出了产气肠杆菌、鲍曼不动杆菌、温和气单胞菌各2例（5.6％），产酸克雷伯菌、铜绿假单胞菌、霍氏肠杆菌、聚团肠杆菌和臭鼻克雷伯菌各1例（2.8％）。实时RS-Carba-R组检测到39个碳青霉烯酶基因，检出最多的酶型为NDM（33/39，84.6％），其次是NDM+KPC（3/39，7.7％），KPC、OXA48及NDM+OXA48各1例（2.6％）。其中6例患者检测出的CRO菌株不同但其携带的碳青霉烯酶基因相同（NDM），3例患者检测出的CRO菌株同时携带两种碳青霉烯酶基因（2例为NDM和KPC，1例为NDM和OXA48）。在本研究中，实时肛拭子Xpert Carba-R检测试剂盒未检出VIM和IMP基因型。

（2）实时肛拭子Xpert Carba-R临床等效性验证：为确定实时肛拭子Xpert Carba-R的准确性，将肛拭子传统培养检出的CRO阳性分离株储存在−80 °C冰柜中，必要时通过复苏其纯化菌落进行Carba-R，再次检测碳青霉烯类抗生素耐药基因。实时RS-Carba-R总体灵敏度、特异度、PPV和NPV的结果见表4。在21例患者中观察到实时RS-Carba-R和纯化菌落Xpert Carba-R的一致阳性结果，被视为真阳性，在143例患者中观察到一致阴性结果，被视为真阴性。其中，11例（6.0％）患者为实时RS-Carba-R阳性但肛拭子传统培养阴性，11例患者的保存标本中，8例标本在培养皿富集碳青霉烯耐药基因PCR后被证实存在碳青霉烯酶基因［NDM（5例），KPC（3例）］，也被视为真阳性；3例标本仍然不一致，因为肉汤培养未确认目标碳青霉烯酶基因而被视为假阳性。1例（0.5％）患者实时RS-Carba-R阴性但肛拭子传统培养阳性，被视为假阴性。8例（4.3％）患者实时RS-Carba-R阴性，纯化菌落Xpert Carba-R也证实为阴性，被视为真阴性，即这8株CRO不携带临床常见的5大酶型。在本研究中，Xpert Carba-R检测试剂盒未检测到VIM和IMP基因。当应用Xpert Carba-R试剂盒生产商推荐的Ct值（38.0个循环）作为临界值时，总体灵敏度、特异度、PPV和NPV分别为96.6％、72.8％、90.6％和88.9％。

**表4:** 纯化菌落Xpert Carba-R和Carba-R的整体性能比较

检测方法	真阳性	假阳性	真阴性	假阴性	灵敏度（％）	特异度（％）	阳性预测值（％）	阴性预测值（％）
纯化菌落Xpert Carba-R	21例	11例	8例	1例	95.5（95％ *CI* 78.2～99.2）	42.1（95％ *CI* 23.2～63.7）	65.6（95％ *CI* 48.3～79.6）	88.9（95％ *CI* 98.2～99.9）
Carba-R	29例	3例	8例	1例	96.6（95％ *CI* 83.3～99.4）	72.8（95％ *CI* 39.1～86.2）	90.6（95％ *CI* 75.8～96.8）	88.9（95％ *CI* 56.5～99.4）

## 讨论

本研究结果显示，同一HSCT患者的CRO肠道定植菌株和CRO-BSI菌株高度一致，具有高度同源性，证明CRO-BSI菌株可能来源于定植菌株，早期发现无症状CRO定植患者对于预警CRO-BSI至关重要。此外，对HSCT患者同时进行RS-培养和实时RS-Carba-R酶型筛查，结果显示，实时肛拭子Xpert Carba-R检测CRO的灵敏度和特异度分别为96.6％和72.8％，与传统的肛拭子培养相比，实时肛拭子Xpert Carba-R检测具有时间短（<1 h）、操作简单快速、检出率高等特点，可替代肛拭子培养。因此，实时肛拭子Xpert Carba-R检测可用于预警CRO-BSI的发生及指导后续CRO-BSI的抗感染治疗，具有临床可行性。

直肠和呼吸道是目前CRO最常见的定植部位，相比于咽拭子筛查，美国疾病控制与预防中心（centers for disease control，CDC）及欧洲临床微生物协会推荐首选的筛查样本是肠道来源样本[13]–[14]，咽拭子采样部位与空气相通，易被杂菌污染，肛拭子标本易于获取，采样部位位于距肛门3～5 cm处，到达位于齿状线上的直肠远端，被认为无杂菌污染、能准确反映患者胃肠道菌群状态。研究显示，CRO的肠道内定植是其后续发生临床感染的独立危险因素，CRO定植患者与其后续发生CRO-BSI高度相关，Demiraslan等[15]研究发现CRO定植患者后续BSI的发生率显著高于非定植患者（33.3％对9.1％，*P*＝0.001），CRO肠道定植可以预警恶性血液病患者发生CRO-BSI[16]，但验证CRO定植菌株与感染菌株同源性的相关研究较少。现阶段不同细菌间常用的同源性研究方法包括MLST、肠道细菌基因间重复序列PCR（ERIC-PCR）、脉冲场凝胶电泳（PFGE）和WGS等[17]–[19]。其中，WGS被认为是分辨率最高的分析方法，可以利用生物信息学分析菌株之间的SNP，从而能更细致地区分菌株之间的差异[20]。本研究采用基质辅助激光解吸电离飞行时间质谱用于菌种鉴定，MLST技术分析菌株序列，WGS技术分析CRO定植菌和BSI菌的耐药基因及进化同源性。WGS显示，同一患者的CRO肠道定植菌株与CRO-BSI菌株的亲缘关系比无关患者更近，此外，同一HSCT患者CRO肠道定植菌株和CRO-BSI菌株携带的碳青霉烯酶基因高度一致，且在感染不同类型CRO的患者中得出一致的结论，证明CRO肠道定植菌株和CRO-BSI菌株具有同源性，CRO肠道定植菌株可以通过从肠道直接迁移到血流中引起BSI，为CRO肠道定植可能预警CRO-BSI提供了理论基础，基于CRO肠道定植菌株的检测结果，可预防CRO-BSI的发生并指导CRO-BSI早期精准治疗。

携带产碳青霉烯酶（CP）基因是CRO耐药的主要机制，CP-CRO的可靠检测对于早期发现CRO肠道定植至关重要。CDC推荐采用基于传统微生物培养的方法检测CP-CRO，但其阳性率低，培养时间长，因此限制了针对碳青霉烯酶等抗生素的治疗效果[21]。一项研究评价了Xpert Carba-R在重症监护病房患者中的检测性能，并报告其灵敏度和特异度分别为100％和96.7％[22]。HSCT患者由于应用大剂量化疗药物和免疫抑制剂、长期使用经验性广谱抗菌药、胃肠道黏膜炎、中性粒细胞减少及长时间住院治疗等，是发生CRO感染的高危人群[23]。既往研究显示，传统肛拭子培养的检出率和灵敏度分别为14.9％和81.8％[7],[24]。为验证实时肛拭子Xpert Carba-R在HSCT患者中的临床应用效能，进行了菌落和补充检测，结果显示，8份肛拭子传统培养阴性的标本后来被Xpert Carba-R鉴定为具有碳青霉烯酶基因，意味着传统肛拭子培养的灵敏度较低，这可能与肠道细菌的丰度较低、采样手法不标准等因素相关。在本研究中，实时肛拭子Xpert Carba-R检测显示的灵敏度和特异度分别为96.6％和72.8％。特异度稍低可能是假阳性结果（可能是由于补充实验的局限性或非特异性结合、标本中细菌负荷较低、样本采集/运输不当导致细菌活力降低或携带碳青霉烯酶基因的微生物不可培养）所致[25]。实时肛拭子Xpert Carba-R的高敏感性意味着它可以及时识别携带CRO定植和碳青霉烯酶基因型的患者，可以替代传统肛拭子培养，预警后续发生CRO-BSI，帮助临床医师采取及时有效的抗感染措施，遏制耐药菌的进一步传播。

本研究仍存在局限性：需要多中心研究进一步充分验证；本研究与CRO的耐药机制和发生环境有关，可能不适用于具有不同耐药机制的CRO和CRO流行率低的其他地区；本研究重点验证CRO的纵向传播，未关注到共轭质粒在不同属、目、门之间的横向传播。后续将扩展研究模式，补充现有研究的不足，对更多变量进行分析，进一步巩固研究结论。

综上所述，在HSCT住院患者中，实时肛拭子Xpert Carba-R通过对CRO耐药菌株进行5种常见碳青霉烯类耐药基因（KPC、NDM、IMP、OXA-48、VIM）检测，可早期准确识别CRO定植患者，有助于临床医师实施耐药菌相关感染防控措施及早期进行精准抢先治疗，对有效降低定植发生率及耐药菌相关死亡率具有重要意义。
